# Gut Microbiota–Bile Acid Axis in Type 2 Diabetes–Associated Gallbladder Diseases: Mechanisms and Therapeutic Potential

**DOI:** 10.3390/metabo16030212

**Published:** 2026-03-21

**Authors:** Qian Zhang, Zhesi Jin

**Affiliations:** School of Medicine, Jiangsu University, Zhenjiang 212013, China; 2222313015@stmail.ujs.edu.cn

**Keywords:** gut microbiota, bile acids, type 2 diabetes mellitus, gallbladder diseases, FXR, TGR5

## Abstract

Gallbladder diseases spanning cholelithiasis, cholecystitis, and gallbladder cancer represent a clinically heterogeneous continuum in which type 2 diabetes mellitus (T2DM) acts as a key metabolic modifier. Conventional models centered on bile supersaturation alone do not sufficiently account for the persistent inflammation and inter-individual variability frequently observed in practice. Here, we synthesize emerging evidence implicating the gut microbiota–bile acid (BA) axis as an integrative mechanism linking metabolic dysregulation, barrier dysfunction, and biliary pathobiology in the diabetic host. Hyperglycemia and insulin resistance, together with impaired mucosal resilience, are associated with shifts in microbial community structure and BA-transforming functions (e.g., bile salt hydrolase and 7α-dehydroxylation), favoring a more hydrophobic BA pool. These changes may disrupt BA receptor signaling, including FXR–FGF15/19 and TGR5-related pathways, thereby amplifying metabolic inflammation, promoting lithogenic bile formation, and impairing gallbladder motility. In parallel, barrier vulnerability may facilitate microbial translocation and LPS-driven immune activation, reinforcing a feed-forward loop that supports the gallstone–inflammation–carcinogenesis trajectory. Translationally, microbiome- and BA-oriented strategies (dietary patterns, bile acid therapeutics, and targeted microbiome modulation) are promising adjuncts, yet precision management should explicitly consider medication- and weight loss–related confounding—particularly with incretin-based therapies—to optimize biliary outcomes across disease stages.

## 1. Introduction

Gallbladder diseases, encompassing a spectrum of pathologies including cholelithiasis, cholecystitis, and gallbladder cancer (GBC), pose a significant global health challenge. Epidemiological evidence indicates that cholelithiasis affects approximately 10–20% of adults worldwide, with nearly 20% of symptomatic patients subsequently progressing to cholecystitis [1,2]. Research indicates that the persistence of gallstones elicits chronic inflammation of the gallbladder mucosa; the synergistic interplay between these factors constitutes a critical driver for the malignant transformation of the gallbladder epithelium [3,4]. As the gravest outcome of this pathological evolution, GBC represents a highly aggressive gastrointestinal malignancy characterized by a dismal prognosis, with a 5-year survival rate remaining below 20% [5].

Notably, diabetes—particularly type 2 diabetes mellitus (T2DM)—constitutes a specific metabolic host context associated with an elevated risk of cholelithiasis and related biliary tract diseases, as well as altered patterns of disease progression [6]. Existing genetic and large-scale cohort studies suggest a robust association between T2DM and cholelithiasis or biliary tract events, with some evidence supporting a potential causal link; yet, heterogeneity regarding outcome definitions and effect sizes persists across studies [7]. This susceptibility likely stems from the distinct pathophysiological milieu of diabetes: diabetic autonomic neuropathy and impaired gallbladder responsiveness to cholecystokinin (CCK) lead to dysmotility and bile stasis, while insulin resistance and hyperinsulinemia drive hepatic cholesterol synthesis and secretion, rendering the bile increasingly lithogenic [8,9]. Furthermore, diabetes is frequently accompanied by compromised intestinal barrier integrity and low-grade systemic inflammation. These conditions may facilitate the translocation of gut-derived lipopolysaccharide (LPS) and magnify inflammatory cascades, such as TLR4–NF-κB signaling, thereby acting as both a trigger and an amplifier in the continuous ‘gallstone–inflammation–carcinoma’ progression [10,11].

Traditional etiological theories predominantly attribute the pathogenesis of gallbladder diseases to physicochemical factors, such as cholesterol supersaturation, bile stasis, and gallbladder dysmotility, providing a crucial theoretical basis for the initial stages of gallstone formation [12]. However, with the accumulation of clinical and omics evidence, this traditional framework has proven insufficient to fully elucidate the significant phenotypic variations among patients, the mechanisms underlying persistent chronic inflammation, and the high heterogeneity in therapeutic responses. Consequently, the research focus has shifted toward more intricate biological networks, wherein the gut microbiota and its metabolites are increasingly recognized to systematically participate in the pathogenesis of gallbladder diseases. Through the remodeling of bile acid metabolism and receptor signaling, as well as the modulation of immune homeostasis and mucosal barriers, these microbial factors drive the evolution of the ‘gallstone–inflammation–carcinogenesis’ continuum [13,14,15,16].

Therefore, positioning type 2 diabetes mellitus (T2DM) as a critical clinical host context, this review systematically elucidates specific alterations in the gut microbiota–bile acid axis within the setting of dysregulated glucose metabolism. We dissect how this axis interacts with hyperglycemia and insulin resistance to drive the ‘gallstone–inflammation–carcinogenesis’ continuum and further evaluate microbiota-targeted therapeutic strategies and their translational prospects. Crucially, we emphasize that evidence interpretation and clinical translation must account for the effects of anti-diabetic medications and weight-loss interventions on gallbladder motility, bile acid metabolism, and the microbiota, thereby minimizing confounding bias to optimize benefit–risk assessments. To support this narrative synthesis, a comprehensive literature search was conducted in the PubMed, Web of Science, and Scopus databases (up to March 2026) using keyword combinations including “gut microbiota”, “bile acids”, “type 2 diabetes mellitus”, and “gallbladder diseases”. Reference selection prioritized recent English-language, peer-reviewed articles—particularly mechanistic studies, omics analyses, and epidemiological cohorts—while incorporating landmark earlier reports necessary to substantiate core pathophysiological concepts.

## 2. Physiological Regulatory Network of the Gut Microbiota–Bile Acid Axis

The gut microbiota–bile acid (BA) axis is a core bidirectional network that maintains BA homeostasis and shapes the initiation and progression of gallbladder diseases. Defining its physiological regulation is essential for understanding how this axis is remodeled in diabetes and how such remodeling contributes to disease pathogenesis.

### 2.1. Bile Acid Synthesis and Enterohepatic Circulation

Bile acid synthesis and enterohepatic circulation are fundamental physiological processes that maintain cholesterol homeostasis and facilitate lipid digestion. For clarity, this process can be considered in three sequential steps: hepatic synthesis, biliary secretion/storage, and intestinal reabsorption with portal return to the liver. This cycle begins with de novo synthesis in hepatocytes, followed by biliary secretion, intestinal reabsorption, and return to the liver, forming an efficient closed loop [17]. Bile acid synthesis occurs almost exclusively in hepatocytes and proceeds primarily through two pathways: the classic (neutral) and the alternative (acidic) routes. The classic pathway accounts for more than 90% of total bile acid production; its rate-limiting step is catalyzed by endoplasmic reticulum cholesterol 7α-hydroxylase (CYP7A1), while cholesterol 12α-hydroxylase (CYP8B1) regulates the relative production of cholic acid (CA) and chenodeoxycholic acid (CDCA). In contrast, the alternative pathway is initiated by mitochondrial sterol 27-hydroxylase (CYP27A1), generates predominantly CDCA, and may serve compensatory roles under specific physiological or pathological conditions [18,19,20,21].

Newly synthesized unconjugated bile acids are subsequently conjugated with glycine or taurine through reactions catalyzed by bile acid–CoA synthetase (BACS) and bile acid–CoA:amino acid N-acyltransferase (BAAT), generating conjugated bile acids with greater water solubility and reduced cytotoxicity. These conjugated bile acids are exported into bile canaliculi by the bile salt export pump (BSEP) and thereby enter bile [22,23,24,25]. During fasting, bile is stored in the gallbladder and concentrated approximately 5–20-fold via epithelial water reabsorption [26]; after feeding, it is released into the duodenum in response to cholecystokinin (CCK), where it emulsifies dietary lipids to facilitate digestion and absorption. The terminal step of this cycle is highly efficient reclamation: ~95% of bile acids are actively reabsorbed in the terminal ileum by the apical sodium-dependent bile acid transporter (ASBT), then transported into the portal circulation via organic solute transporter α/β (OSTα/OSTβ), and finally taken up by hepatocytes through sinusoidal transporters such as the Na^+^-taurocholate cotransporting polypeptide (NTCP) [27,28,29,30]. This closed loop typically recirculates ~6–10 times per day, allowing an approximately 3 g bile acid pool to be efficiently reused; only ~5% is lost in feces daily and is replenished by hepatic de novo synthesis, thereby maintaining systemic homeostasis [31,32].

### 2.2. Microbial Transformation of Bile Acids and Regulatory Pathways

The gut microbiota shapes bile acid (BA) metabolism through two major and interconnected processes: direct enzymatic biotransformation of bile acids and indirect modulation of host bile acid signaling pathways. First, microbial bile salt hydrolases (BSHs) deconjugate conjugated BAs into unconjugated species; these are then further modified by hydroxysteroid dehydrogenases (HSDHs) and 7α-dehydroxylation–related enzyme systems, yielding secondary BAs such as deoxycholic acid (DCA) and lithocholic acid (LCA) [33,34,35]. This biotransformation expands BA chemical diversity and alters key physicochemical features of the BA pool.

Beyond direct biotransformation, the microbiota also modulates BA homeostasis by influencing host signaling pathways, with the farnesoid X receptor (FXR)–fibroblast growth factor 15/19 (FGF15/19) axis serving as a central regulatory node. In murine models, microbial deconjugation and related processes shift BA composition—including FXR-antagonistic species such as tauro-β-muricholic acid (T-βMCA)—thereby regulating intestinal FXR activity and, through feedback, hepatic BA synthesis [36,37,38]. In this context, intestinal FXR functions as a key upstream sensor of luminal BA composition and regulates hepatic CYP7A1 primarily via FGF15/19-mediated feedback signaling. Of note, the human BA pool differs from that of rodents (muricholic acids are not dominant constituents, and FXR activity is primarily governed by chenodeoxycholic acid (CDCA) and its derivatives); nevertheless, microbial control over BA conjugation status and hydrophobicity, and its downstream impact on FXR signaling, appears to be conserved across species [17]. Moreover, microbiota-derived secondary BAs can activate the G protein–coupled BA receptor TGR5, among other pathways, thereby mediating broader metabolic effects, including anti-inflammatory signaling [39,40].

### 2.3. Regulation of Gut Microbial Composition by Bile Acids

The crosstalk between the gut microbiota and bile acids (BAs) is inherently bidirectional: the microbiota reshapes the BA pool, whereas BAs, as both selective ecological pressures and signaling molecules, in turn shape microbial community structure and function. BAs influence the microbiota primarily through two routes. First, more hydrophobic BAs exert detergent-like antimicrobial activity, disrupting bacterial membranes and interfering with key metabolic processes, thereby suppressing the growth of susceptible taxa [33,41]. Second, BAs can modulate microbial colonization indirectly by activating host signaling pathways—most notably intestinal FXR—to regulate innate immune gene programs and reshape the mucosal immune milieu [42,43,44,45].

A range of physiological, pathological, and pharmacological observations supports the role of BAs as determinants of microbial community composition. For example, in high-fat diet–associated metabolic models, shifts in BA profiles often coincide with microbial community restructuring, and supplementation with tauroursodeoxycholic acid (TUDCA) can partially reverse dysbiosis [46,47]. In FXR-deficient mice, alterations in the BA pool markedly remodel the gut microbiome, underscoring feedback regulation of microbial ecology by host receptor signaling [48,49]. Moreover, microbiome changes observed following treatment with FXR agonists (e.g., obeticholic acid) further suggest that BA concentration and composition constitute important ecological determinants of gut microbial niches [50,51].

Taken together, this physiological regulatory network helps define BA pool composition, BA receptor signaling strength, and the stability of the intestinal ecological niche. Against this physiological backdrop, diabetes-associated hyperglycemia, insulin resistance, and low-grade inflammation may amplify vulnerable nodes along the gut microbiota–BA axis, thereby shifting the system from homeostatic regulation toward pathological remodeling. This framework motivates the subsequent discussion of diabetes-associated alterations and their relevance to gallbladder disease progression.

## 3. Diabetes-Associated Remodeling: Pathological Specificity of the Gut Microbiota–Bile Acid Axis

In the diabetic host, this bidirectional regulatory network that maintains physiological homeostasis can undergo marked pathological remodeling. Hyperglycemia and insulin resistance may disrupt bile acid metabolism by reshaping specific microbial functional modules and, through receptor-mediated feedback loops, further amplify metabolic and immune dysregulation, thereby providing a favorable pathophysiological context that may facilitate the initiation and progression of gallbladder diseases.

### 3.1. Diabetes-Associated Dysbiosis and Microbial Functional Modules for Bile Acid Metabolism

**Figure 1:**
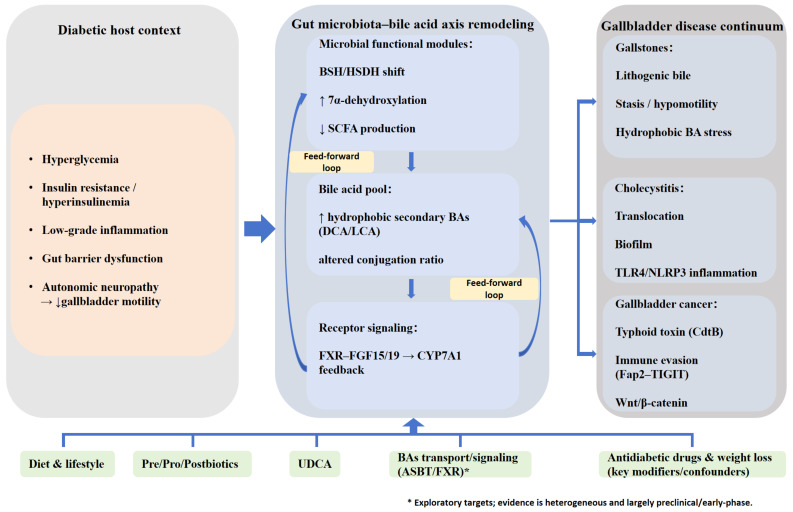
Mechanistic framework and intervention nodes of the gut microbiota–bile acid axis in diabetes-associated gallbladder diseases. The diabetic host context drives shifts in microbial functional modules and reshapes the bile acid pool, perturbing receptor signaling and promoting the gallbladder disease continuum. The figure highlights how T2DM-associated metabolic dysregulation, immune activation, and barrier impairment interact with microbiota-mediated bile acid remodeling to influence disease progression from cholelithiasis and cholecystitis to gallbladder cancer.

Compared with healthy individuals, the gut microbiota in patients with type 2 diabetes mellitus (T2DM) frequently exhibits a distinct dysbiotic pattern, typified by reduced abundance of butyrate-producing taxa (e.g., Faecalibacterium prausnitzii and Roseburia) and a relative expansion of opportunistic pathogens (e.g., Escherichia coli and Desulfovibrio) [52,53,54]. These taxonomic shifts are closely linked to dysregulation of microbial functional modules governing bile acid metabolism. In T2DM, changes in bile salt hydrolase (BSH) activity may disrupt the deconjugation of conjugated bile acids, thereby shaping downstream secondary bile acid production. In parallel, an increased abundance of bacteria with 7α-dehydroxylation capacity has been associated with greater formation of more hydrophobic secondary bile acids, such as deoxycholic acid (DCA), and may contribute to their accumulation under diabetic conditions (Figure 1) [44,55]. In addition, reduced short-chain fatty acid (SCFA) production may lower luminal acidity, thereby further influencing bile acid solubility and intestinal reabsorption, ultimately shifting the bile acid pool toward greater overall hydrophobicity and an imbalanced composition [56,57].

### 3.2. Diabetes-Associated Bile Acid Profiles and Metabolic Coupling via FXR–FGF15/19 and TGR5–GLP-1

Bile acid (BA) profiles remodeled under diabetic conditions are not merely downstream consequences of metabolic disturbance; they also act as signaling mediators that help regulate host glucose and lipid homeostasis [33,58,59]. Along the gut–liver axis, shifts in the balance between BA agonists and antagonists can perturb intestinal farnesoid X receptor (FXR) activity, leading to altered secretion of fibroblast growth factor 15/19 (FGF15/19) [60,61]. This may attenuate negative-feedback suppression of hepatic CYP7A1, thereby promoting dysregulated BA synthesis and impaired cholesterol metabolism [33,62]. In parallel, changes in BA composition may reduce the sensitivity of TGR5 signaling and blunt TGR5-mediated glucagon-like peptide-1 (GLP-1) secretion from enteroendocrine L cells. Given the role of GLP-1 in postprandial insulin secretion, gastric emptying, and gallbladder motility, dysfunction of the TGR5–GLP-1 axis may exacerbate glycemic dysregulation and may be linked to reduced gallbladder motility (Figure 1) [63,64]. Consequently, in diabetes, fluctuations in the microbiota–BA axis may be amplified at key nodes such as FXR and TGR5, reinforcing a feed-forward loop that couples dysbiosis with BA metabolic disruption and worsening glucose–lipid metabolism [33,65]. Overall, T2DM appears to reshape microbial bile acid transformation through coordinated alterations in glycemic status, insulin resistance, intestinal microbial ecology, and host bile acid signaling pathways, thereby creating a metabolic milieu that may predispose to downstream gallbladder dysfunction.

### 3.3. Diabetes-Related Host Factors Drive Axis Dysfunction Toward the Gallbladder Disease Continuum

This systemic disruption of the microbiota–bile acid axis ultimately converges on the gallbladder as a target organ, manifesting as stage-specific pathogenic drivers. First, metabolic dysregulation can shift the cholesterol-to-bile acid balance in bile, generating a more lithogenic milieu; concurrently, diabetes-associated autonomic neuropathy may impair gallbladder contractility, further promoting bile retention and creating physicochemical conditions favorable for gallstone formation [66]. Second, chronic inflammatory pathways can be persistently engaged: gut barrier dysfunction facilitates the entry of gut-derived endotoxin (e.g., lipopolysaccharide, LPS) and hydrophobic secondary bile acids into the hepatobiliary system, thereby activating local TLR4 signaling and the NLRP3 inflammasome and potentially facilitating the transition from asymptomatic cholelithiasis to acute or chronic cholecystitis (Figure 1) [67,68,69]. Finally, under sustained inflammatory stress—together with hyperinsulinemia, hyperglycemia, and exposure to cytotoxic hydrophobic bile acids—gallbladder epithelial cells experience ongoing oxidative stress and DNA damage, which may accelerate fibrotic remodeling and contribute to a pro-tumorigenic microenvironment associated with malignant transformation [70,71,72]. Building on this framework, the next section examines stage-specific manifestations across cholelithiasis, cholecystitis, and gallbladder cancer.

## 4. Gallbladder Disease Continuum in the Diabetic Host

Compared with the general population, the diabetic host is characterized by metabolic dysregulation, low-grade inflammation, and impaired mucosal barrier resilience, which may heighten vulnerability to gut microbiota–bile acid axis perturbations. This host context can favor lithogenic bile, sustain chronic inflammation, and increase the risk of malignant transformation across disease stages. Collectively, these features may contribute to the increased burden and clinical heterogeneity of gallbladder diseases in diabetes and support a mechanism-informed, stage-stratified approach to integrated management.

### 4.1. Cholelithiasis

#### 4.1.1. Dysbiosis-Related Signatures and Pathogenic Mechanisms

Gallstones are a key initiating factor for adverse biliary outcomes, including acute cholecystitis, biliary pancreatitis, and ultimately gallbladder cancer. However, traditional etiologic models—largely centered on physicochemical disturbances of bile—remain insufficient to explain the marked clinical heterogeneity and accompanying chronic inflammation observed across patients [73,74]. Emerging evidence suggests that gut microbial shifts and altered metabolic capacity may contribute to lithogenic bile by perturbing bile acid homeostasis, cholesterol handling, and mucosal immune tone, thereby offering a mechanistic framework to account for this heterogeneity [75].

Studies have linked cholelithiasis to gut microbial dysbiosis, commonly characterized by reduced α-diversity and a shifted β-diversity structure [74,76]. Taxonomically, several reports describe depletion of protective commensals—particularly butyrate-associated taxa such as Faecalibacterium and Roseburia—alongside enrichment of inflammation-associated communities, including expansion of Proteobacteria and opportunistic pathogens [77,78,79]. These alterations may compromise barrier integrity and anti-inflammatory capacity and may perturb intestinal handling of cholesterol and bile acids [80]. In parallel, relative enrichment of opportunistic taxa (e.g., Escherichia–Shigella and Klebsiella) may contribute to lithogenesis through multiple pathways: (i) increased abundance of bacteria with 7α-dehydroxylation capacity may reshape enterohepatic cycling and increase the proportion of hydrophobic secondary bile acids; (ii) hydrogen sulfide produced by Desulfovibrio may interfere with hepatobiliary regulation of bile acid synthesis and disrupt cholesterol homeostasis; and (iii) elevated β-glucuronidase activity may promote bilirubin deconjugation, facilitating nucleation of pigment stones [15,81,82,83].

In the diabetic host, these lithogenic pathways may interact across metabolic, neurogenic, and microbiome-related dimensions. Diabetes-associated autonomic neuropathy can impair gallbladder contraction and emptying, thereby promoting bile retention. Insulin resistance–driven increases in hepatic cholesterol output can render bile more supersaturated, providing substrate for nucleation and crystallization. In parallel, hyperglycemia-associated barrier vulnerability and a background of low-grade inflammation may lower the activation threshold of inflammatory cascades such as LPS–TLR4–NF-κB, further exacerbating mucosal inflammation and gallbladder hypomotility [11,66,84]. Collectively, gallstone formation in diabetes may be more strongly shaped by a composite mechanism in which metabolic dysregulation and chronic low-grade inflammation reinforce one another.

#### 4.1.2. Targeted Therapeutic Strategies

Growing recognition of the gut microbiota’s role in cholelithiasis has motivated an integrated intervention framework aimed at restoring microbial homeostasis and optimizing the bile acid pool. Conventional dissolution therapies (e.g., UDCA/CDCA) may confer benefits beyond physicochemical litholysis by reshaping bile acid composition and coinciding with shifts in gut microbial ecology [85,86]. As more direct microbiome-modulating approaches, selected probiotics (e.g., specific *Lactobacillus* strains) have been shown in experimental settings to reduce lithogenic propensity by influencing bile acid metabolism and receptor signaling. Dietary interventions—such as a Mediterranean-style pattern rich in fiber and low in saturated fat—may likewise lower gallstone risk by increasing microbial diversity and improving metabolic status [87,88,89]. Emerging modalities, including fecal microbiota transplantation (FMT) and biomaterials designed to target biofilms or enhance local delivery, remain exploratory but may offer adjunct options for refractory or high-recurrence-risk populations [80,90,91]. In mechanistic terms, FXR agonists and TGR5-directed modulators may also hold therapeutic relevance by restoring BA signaling balance and improving metabolic–biliary coupling; however, their direct efficacy in diabetes-associated gallbladder disease remains to be established.

In patients with type 2 diabetes mellitus (T2DM) complicated by cholelithiasis, both translational research and clinical decision-making should explicitly consider the potential effects of antidiabetic medications and weight change on gallbladder and biliary events. Systematic reviews and meta-analyses suggest that glucagon-like peptide-1 receptor agonists (GLP-1RAs) provide substantial weight-loss benefits yet, in certain settings, are associated with an increased risk of gallbladder and biliary events; by contrast, the risk signals for dipeptidyl peptidase-4 inhibitors (DPP-4is) and sodium–glucose cotransporter-2 inhibitors (SGLT2is) remain heterogeneous across studies and should be interpreted in the context of population characteristics, comparator therapies, dose, and treatment duration [92,93,94] (Table 1). While clinical studies have helped define the risk profiles of these glucose-lowering therapies, the underlying mechanisms involving the microbiota–bile acid axis are still supported predominantly by preclinical experimental evidence, highlighting the need for further translational validation in humans. In addition, observational studies have linked metformin use to a lower risk of gallstones, potentially through improvements in insulin resistance and modulation of the gut microbiota, although prospective confirmation is still needed [95,96].

After metabolic/bariatric surgery, rapid weight loss and remodeling of the gut–bile acid ecosystem may increase short-term gallstone risk. Available evidence suggests that prophylactic UDCA can reduce gallstone incidence in selected high-risk individuals, but optimal patient selection and dosing regimens remain to be refined and validated. Overall, management of cholelithiasis in T2DM should balance metabolic benefits against biliary risks using an individualized risk assessment, with explicit consideration of medication- and weight loss–related confounding factors [97,98,99,100].

### 4.2. Cholecystitis

#### 4.2.1. Dysbiosis-Related Signatures and Pathogenic Mechanisms

Unlike cholelithiasis, which is largely driven by physicochemical disturbances of bile, cholecystitis more prominently reflects an inflammatory process shaped by bacterial translocation, immune activation, and disruption of the gallbladder barrier [101,102]. Emerging evidence suggests that the gut microbiota can influence inflammatory responses through immunomodulatory effects and may also serve as a pathogen reservoir for biliary infection via gut–biliary routes [103,104]. At the microbial level, patients with cholecystitis often exhibit more pronounced dysbiosis, characterized by enrichment of Gram-negative and opportunistic taxa, depletion of commensals, and an increased lipopolysaccharide (LPS) burden [105,106,107]. In addition, cultivable bacteria in bile and biofilm formation on stones or mucosa support a role for microbial persistence in recurrent or chronic infection [108,109].

In people with diabetes, impaired innate immunity (including reduced neutrophil chemotaxis, phagocytosis, and bactericidal activity) together with microvascular disease–related hypoperfusion may compromise clearance of ascending biliary infection and amplify inflammatory responses, thereby increasing the risk of severe disease or complications [110,111].

#### 4.2.2. Targeted Therapeutic Strategies

Standard management of cholecystitis relies on antimicrobial therapy and, when indicated, surgical or interventional source control. As adjunctive approaches, microbiome-targeted interventions may be of potential value in selected settings (e.g., recurrent disease, chronicity, or postoperative recovery), although the evidence base and the most appropriate target populations remain to be defined. In the acute phase, in addition to antibiotics covering common pathogens, early nutritional support and glycemic control may help preserve mucosal barrier function and systemic homeostasis [112,113]. During recovery, strategies such as postbiotics (e.g., butyrate) combined with hydrophilic bile acids (e.g., UDCA) may be considered to support barrier repair and optimize bile acid profiles, but their efficacy and safety require prospective validation [114]. In the perioperative period or during acute inflammation, use of glucose-lowering agents that may affect gastrointestinal and gallbladder motility (e.g., GLP-1RAs) should be individualized according to clinical status, patient tolerance, and biliary risk [92].

### 4.3. Gallbladder Cancer

#### 4.3.1. Dysbiosis-Related Signatures and Pathogenic Mechanisms

Gallbladder cancer (GBC) is often viewed as a terminal consequence of long-standing chronic inflammation; however, emerging evidence suggests that microbiome-associated oncogenic dysbiosis may also contribute to its initiation and progression [115,116]. In some studies, GBC-related specimens show enrichment of specific taxa (e.g., *Fusobacterium nucleatum* and *Salmonella typhi*), which may influence tumor development through genotoxicity, activation of oncogenic signaling pathways, and remodeling of the immune microenvironment [117,118]. Notably, evidence implicating *Helicobacter* species and related virulence factors in biliary samples largely derives from detection-based associations and mechanistic inference; causality remains to be established in rigorously stratified cohorts and functional studies [119,120].

In the context of diabetes, hyperinsulinemia/IGF-1 axis activation and chronic low-grade inflammation may act in concert to increase the burden of DNA damage. Concurrently, a shift toward a more hydrophobic bile acid profile (e.g., elevated deoxycholic acid, DCA) may be linked to activation of pro-oncogenic signaling, thereby reinforcing a pro-tumorigenic niche through coupled metabolic, immune, and microbiome perturbations [121,122].

#### 4.3.2. Targeted Therapeutic Strategies

Microbiome-informed interventions—including bacteriophage therapy, nano-enabled delivery platforms, engineered probiotics/postbiotics, and fecal microbiota transplantation (FMT) or defined synthetic consortia—remain largely at the preclinical stage in GBC, and their feasibility, safety, and efficacy need to be evaluated in GBC-specific populations [123,124]. Although FMT has been explored as a strategy to enhance responses to immunotherapy in other tumor types, evidence in GBC remains limited; extrapolation should therefore be cautious, and dedicated validation studies are warranted [125].

For patients with GBC and comorbid diabetes, metabolic factors should be incorporated as key effect modifiers in overall clinical assessment. When selecting glucose-lowering therapy, agents such as metformin may be considered given their potential anti-tumor and microbiome-modulating effects (Table 1), but prospective studies are required to confirm clinical benefit in this setting [126,127]. Key microbial signatures, microbiota–bile acid axis mechanisms, and diabetes-specific amplifying factors across cholelithiasis, cholecystitis, and GBC are summarized in Table 2.

## 5. Conclusions and Perspectives

Synthesizing the available evidence, this review suggests that the gut microbiota–bile acid (BA) axis may serve as a key integrative network linking metabolic, immune, and mucosal barrier pathways and may participate in the evolution of the gallbladder disease continuum—from cholelithiasis and cholecystitis to gallbladder cancer. In type 2 diabetes mellitus (T2DM), hyperglycemia and insulin resistance, together with low-grade inflammation and impaired barrier integrity, may cooperatively reshape microbial BA-transforming capacity and alter BA pool composition. These shifts can perturb BA receptor signaling—particularly the FXR–FGF15/19 axis and TGR5-dependent pathways—thereby amplifying metabolic and inflammatory disturbances and increasing susceptibility to gallbladder dysmotility, lithogenic bile formation, and disease progression.

From a translational perspective, interventions targeting the microbiome and BA signaling (e.g., dietary optimization, pre-/pro-/postbiotics, UDCA, and pharmacologic modulation of BA transport or receptor pathways) represent potential adjunct strategies for diabetes-associated gallbladder diseases. However, interpretation of therapeutic outcomes in T2DM should systematically account for the effects of glucose-lowering medications and weight-loss interventions on gallbladder motility, BA metabolism, and microbial ecology to reduce confounding and refine benefit–risk assessment, particularly in the context of GLP-1–based therapies or rapid weight reduction.

Key knowledge gaps remain, including limited causal inference, heterogeneity in sampling and BA profiling pipelines, and an incomplete mapping between taxonomic shifts and functional outputs. Future priorities include diabetes-stratified prospective cohorts (e.g., HbA1c, disease duration, and complication burden) with medication stratification, integrated with BA metabolomics and multi-omics to enable causal modeling. Greater emphasis is also needed on stage-specific characterization across the gallbladder disease continuum, particularly to distinguish microbial and BA signatures associated with cholelithiasis, cholecystitis, and gallbladder cancer. In parallel, translational studies and well-designed interventional trials are needed to determine whether microbiota- or BA-targeted strategies can achieve reproducible clinical benefit, while also clarifying their safety, durability, and optimal patient selection in T2DM. Well-designed, stage-specific clinical trials with multidimensional endpoints—including clinical outcomes, BA signatures, inflammatory markers, and gallbladder motility metrics—will be essential to translate mechanistic insights into precision, stratified management for this high-risk comorbid population.

## Figures and Tables

**Table 1:** Clinical impact of glucose-lowering therapies and weight-loss interventions on gallbladder/biliary risk and their proposed mechanisms via the gut microbiota–bile acid axis.

Intervention	Clinical Impact on Gallbladder/Biliary Risk	Proposed Mechanisms (Predominantly Preclinical Evidence)	References
GLP-1 receptor agonists (e.g., semaglutide, liraglutide)	Increased (risk positively associated with dose and treatment duration)	• Inhibits gastric emptying and gallbladder contraction (↓ CCK sensitivity) • Rapid weight loss → cholesterol supersaturation • Interferes with bile acid signaling via TGR5-related feedback	[92,93,94]
Biguanides (metformin)	Potentially protective (long-term use may reduce gallstone risk)	• insulin resistance, reducing hepatic Improves cholesterol synthesis • Modulates specific taxa (e.g., ↑ Akkermansia) • Increases hydrophilicity of the bile acid pool	[95,96]
DPP-4 inhibitors (e.g., sitagliptin)	Moderately increased or neutral (overall risk lower than GLP-1RAs)	• Alters incretin levels, mildly affecting biliary motility • Less pronounced effects on body weight	[92,93,94]
SGLT2 inhibitors (e.g., empagliflozin)	Neutral (no consistent signal of increased risk)	• Primarily renal effects (osmotic diuresis) • Does not directly disrupt gallbladder motility or the bile acid axis	[92,93,94]
Metabolic/bariatric surgery (e.g., RYGB, SG)	Very high risk (especially in the early postoperative period)	• Rapid weight loss mobilizes large amounts of tissue cholesterol • Anatomical reconstruction markedly alters enterohepatic circulation and the gut ecosystem	[97,98,99,100]
Prophylactic UDCA therapy (with weight-loss interventions)	Reduced risk (significantly lowers gallstone formation)	• Supplements hydrophilic bile acids and counteracts hydrophobic bile acid toxicity • Corrects bile lithogenicity index	[97,98,99,100]

Abbreviations: CCK, cholecystokinin; DPP-4, dipeptidyl peptidase-4; GLP-1, glucagon-like peptide-1; GLP-1RA, glucagon-like peptide-1 receptor agonist; RYGB, Roux-en-Y gastric bypass; SG, sleeve gastrectomy; SGLT2, sodium-glucose cotransporter 2; TGR5, Takeda G protein-coupled receptor 5; UDCA, ursodeoxycholic acid. Note: To distinguish the levels of evidence more clearly, the “Clinical Impact on Gallbladder/Biliary Risk” column summarizes findings from human studies, including epidemiological cohorts and meta-analyses, whereas the “Proposed Mechanisms (Predominantly Preclinical Evidence)” column primarily reflects translational and preclinical evidence derived from animal and in vitro studies.

**Table 2:** Key microbial signatures, microbiota–bile acid axis mechanisms, and diabetes-specific amplifying factors across the gallbladder disease continuum.

Disease Stage	Key Microbial Signatures	Core Mechanisms via Gut-Biliary Axis	Diabetes-Specific Amplifying Factors
Cholelithiasis	↓ Beneficial commensals: Faecalibacterium, Roseburia ↑ Opportunistic pathogens: Escherichia-Shigella, Desulfovibrio	• Remodeling of BA transformation: Enhanced BSH/7α-dehydroxylation activity → ↑ Hydrophobic BAs (e.g., DCA) → Disrupted FXR negative feedback • Nucleation: ↑ β-glucuronidase → Bilirubin deconjugation → Pigment stone nucleation • Metabolic disruption: H_2_S production (Desulfovibrio) → Interference with cholesterol-BA homeostasis	• ↑ Bile lithogenicity: Insulin resistance drives excessive hepatic cholesterol secretion • ↓ Gallbladder motility: Autonomic neuropathy leads to impaired emptying and stasis • “Permissive” leaky gut: Compromised barrier integrity → ↓ Threshold for LPS-triggered inflammation
Cholecystitis	↑ Gram-negative bacteria (Dominance): Enterobacteriaceae (e.g., E. coli, Klebsiella), Pseudomonas ↑ Others: Enterococcus	• Bacterial translocation: Gut-derived bacteria → Retrograde migration via compromised barrier → Colonization of the hepatobiliary system • Biofilm formation: Acts as a "bacterial shield" on stone/mucosal surfaces → Persistent infection and antimicrobial resistance • Amplification of inflammatory cascades: LPS → Activation of TLR4/NF-κB and NLRP3 inflammasomes	• Immune paralysis: ↓ Neutrophil chemotaxis and phagocytosis → Impaired clearance of retrograde infections • Microenvironmental deterioration: Microvascular complications → Local ischemia/hypoxia → Facilitates anaerobic bacterial growth • Glucotoxicity: Directly promotes the release of pro-inflammatory cytokines
GBC	↑ Pro-carcinogenic microbiota (Specific): Salmonella Typhi (carrier state), Helicobacter spp. (e.g., H. bilis), Fusobacterium nucleatum	• Genotoxicity: Toxin secretion (e.g., typhoid toxin CdtB) → DNA double-strand breaks • Synergistic oncogenic signaling: Hydrophobic BAs (DCA) + Inflammation → Activation of Wnt/β-catenin pathway • Immune evasion: F. nucleatum utilizes the Fap2–TIGIT axis → Inhibition of NK cell-mediated cytotoxicity	• “Second hit”: Hyperinsulinemia/activation of the IGF-1 axis → Potent mitogenic effects • Oxidative stress: Systemic chronic low-grade inflammation → ↑ DNA damage burden • Microenvironmental remodeling: Pro-carcinogenic metabolic environment → Accelerates malignant transformation

Abbreviations: BA, bile acid; BSH, bile salt hydrolase; CdtB, cytolethal distending toxin subunit B; DCA, deoxycholic acid; FXR, farnesoid X receptor; GBC, gallbladder cancer; H_2_S, hydrogen sulfide; IGF-1, insulin-like growth factor 1; LPS, lipopolysaccharide; NF-κB, nuclear factor kappa B; NK, natural killer; NLRP3, NOD-like receptor family pyrin domain-containing 3; TIGIT, T cell immunoreceptor with Ig and ITIM domains; TLR4, Toll-like receptor 4; Wnt, Wingless-related integration site. ↑ indicates an increase, enhancement, or enrichment; ↓ indicates a decrease, impairment, or depletion.

## Data Availability

No new data were created or analyzed in this study. Data sharing is not applicable to this article.

## Abbreviations

ASBT	Apical sodium-dependent bile acid transporter
BA	Bile acid
BAAT	Bile acid–CoA:amino acid N-acyltransferase
BACS	Bile acid–CoA synthetase
BSEP	Bile salt export pump
BSH	Bile salt hydrolase
CA	Cholic acid
CCK	Cholecystokinin
CDCA	Chenodeoxycholic acid
CoA	Coenzyme A
CdtB	Cytolethal distending toxin subunit B (typhoid toxin component)
CYP7A1	Cholesterol 7α-hydroxylase
CYP8B1	Cholesterol 12α-hydroxylase
CYP27A1	Sterol 27-hydroxylase
DCA	Deoxycholic acid
DPP-4	Dipeptidyl peptidase-4
DPP-4i	Dipeptidyl peptidase-4 inhibitor
FGF15/19	Fibroblast growth factor 15/19 (FGF15 in mice; FGF19 in humans)
FMT	Fecal microbiota transplantation
FXR	Farnesoid X receptor
GBC	Gallbladder cancer
GLP-1	Glucagon-like peptide-1
GLP-1RA	Glucagon-like peptide-1 receptor agonist
HbA1c	Glycated hemoglobin A1c
H_2_S	Hydrogen sulfide
HSDH	Hydroxysteroid dehydrogenase
IGF-1	Insulin-like growth factor 1
LCA	Lithocholic acid
LPS	Lipopolysaccharide
NF-κB	Nuclear factor kappa B
NK cell	Natural killer cell
NLRP3	NOD-like receptor family pyrin domain-containing 3
NTCP	Na^+^-taurocholate cotransporting polypeptide
OSTα/OSTβ	Organic solute transporter alpha/beta
RYGB	Roux-en-Y gastric bypass
SCFA	Short-chain fatty acid
SG	Sleeve gastrectomy
SGLT2	Sodium–glucose cotransporter 2
SGLT2i	Sodium–glucose cotransporter 2 inhibitor
T2DM	Type 2 diabetes mellitus
T-βMCA	Tauro-β-muricholic acid
TGR5	Takeda G protein–coupled receptor 5 (also known as GPBAR1)
TIGIT	T cell immunoreceptor with Ig and ITIM domains
TLR4	Toll-like receptor 4
TUDCA	Tauroursodeoxycholic acid
UDCA	Ursodeoxycholic acid
Wnt	Wingless-related integration site signaling pathway
